# Maternal separation-induced changes in breast milk fatty acid composition are associated with altered gut microbiota and serotonergic gene expression in neonatal mice

**DOI:** 10.1186/s43556-026-00508-2

**Published:** 2026-06-28

**Authors:** Eman A. Mady, Hussein M. El-Husseiny, Jun Kambe, Sora Masukado, Shiho Miyata, Taiki Terajima, Hong Liu, Ryo Inoue, Chunmei Li, Yuki Yamamoto, Kentaro Nagaoka

**Affiliations:** 1 Laboratory of Veterinary Physiology, Cooperative Department of Veterinary Medicine, Tokyo University of Agriculture and Technology, Tokyo, 183-8509 Japan; 2 Department of Animal Hygiene, Behavior, and Management, Faculty of Veterinary Medicine, Benha University, Moshtohor, Toukh, Elqaliobiya 13736 Egypt; 3 Institute of Global Innovation Research, Tokyo University of Agriculture and Technology, 3-8-1 Harumi-Cho, Fuchu-Shi, Tokyo, 183-8538 Japan; 4 Department of Surgery, Anesthesiology, and Radiology, Faculty of Veterinary Medicine, Benha University, Moshtohor, Toukh, Elqaliobiya Egypt; 5 Laboratory of Animal Science, Department of Applied Biological Science, Setsunan University, Osaka, Japan; 6 College of Animal Science and Technology, Nanjing Agricultural University, Nanjing, China

**Keywords:** Stress, Maternal separation, Microbiota, Metabolome, Gut-brain axis

## Abstract

Maternal separation (MS) is a widely used model of early-life stress, yet the biological pathways transmitting maternal stress signals to offspring remain incompletely understood. Breast milk represents a primary biological interface between mother and infant, and stress-induced changes in milk composition may influence offspring development through the gut microbiota–brain axis. Here, we investigated whether MS alters the breast milk metabolome and whether such changes are associated with offspring gut microbiota composition, serum metabolite profiles, and serotonin-related gene expression in the prefrontal cortex. Using an integrated multi-omics approach in mice, we found that MS dams had elevated myristic acid and ethanolamine levels in breast milk. MS offspring exhibited reduced *A. muciniphila* abundance in the cecal microbiota, elevated myristic acid and decreased inosine in cecal contents, decreased inosine in serum, and reduced *Tph2* and *Htr1b* expression in the prefrontal cortex. Oral myristic acid supplementation in neonatal pups reduced *A. muciniphila* abundance, and inosine supplementation selectively increased *Htr1a* expression. Spearman correlation analyses using paired individual data revealed a negative association between cecal myristic acid and cecal inosine levels, and a positive correlation between cecal inosine and prefrontal *Tph2* expression, supporting quantitative links among these components. Collectively, these findings identify coordinated associations among maternal stress-induced milk fatty acid changes, offspring gut microbiota, microbial metabolites, and serotonin-related gene expression, providing an exploratory framework for understanding milk-mediated effects on the developing gut–brain axis.

**Supplementary Information:**

The online version contains supplementary material available at 10.1186/s43556-026-00508-2.

## Introduction

The early postnatal period is characterized by extensive brain development. During this period, external factors such as early life stress (ELS) can substantially impact brain development and function, with effects that may persist throughout life [1]. ELS is a determinant of vulnerability to diverse disorders, including dysfunction of the brain and gut [2]. Because maternal care is critical for infant brain growth and dam health, ELS can disrupt the dam-pup connection, leading to neurobiological alterations in the offspring [3]. In this context, maternal separation (MS) has been widely used as an experimental model of ELS and has been shown to contribute to the development of neuropsychiatric disorders [4]. Repeated early postnatal separation of pups from dams evokes emotional stress in the mother [5], which can alter maternal behavior and disrupt the quality of maternal care, thereby indirectly influencing offspring development [6]. While the behavioral consequences of MS have been extensively studied, the biological pathways by which maternal stress signals are transmitted from the dam to the offspring remain incompletely understood. One plausible route is breast milk, which represents a primary biological interface between mother and infant. Beyond its nutritional role, breast milk contains a wide array of bioactive components capable of shaping early physiological development [7, 8]. Maternal stress may alter milk composition, thereby modifying the biological information conveyed to the offspring; however, how such changes influence downstream developmental processes remains unclear [9].

Early postnatal development of the gut microbiota (GM) is increasingly recognized as a key factor in shaping brain development through the gut–brain axis (GBA) [10]. The GM, which refers to the ecological community of microorganisms, plays a critical role in conserving the intestinal mucosal barrier and avoiding colonization by pathogenic microorganisms [9]. Moreover, the GM can regulate brain function through distinct pathways. The relationship between GM and mental health has emerged as a rapidly growing area of research. The GBA represents a bidirectional interaction between the gut and brain. Diverse pathways are engaged in the GBA, including the autonomic (via the vagus nerve) nervous system, hypothalamic–pituitary–adrenal (endocrine), and immune pathway [9, 11, 12]. Importantly, GM-derived metabolites have been shown to influence brain function and behavior [13], suggesting that alterations in early microbial colonization or metabolic activity may have long-term neurodevelopmental consequences.

One neurobiological system sensitive to early-life environmental signals is the serotonergic system. Serotonin (5-hydroxytryptamine, 5-HT) plays a central role in brain maturation, emotional regulation, and behavior [14, 15]. Accumulating evidence indicates that gut-derived signals can modulate serotonergic pathways via the GBA, in part through changes in microbial composition and metabolite production [16]. Notably, maternal psychological stress during lactation has been reported to alter the fatty acid composition of breast milk, including shifts in saturated fatty acid profiles [17, 18]. Saturated fatty acids are known to possess antimicrobial properties that can inhibit bacterial growth and biofilm formation [19, 20]. Given that breast milk is a primary determinant of early microbial colonization in the neonatal gut [7, 9], stress-induced alterations in milk fatty acid content could plausibly influence the establishment of the gut microbiota during this critical developmental window. Among gut commensals, mucus-resident species such as *Akkermansia muciniphila* are considered particularly sensitive to changes in the intestinal environment, including dietary lipid composition [21]. However, whether maternal stress-induced alterations in breast milk can shape GM development and subsequently influence serotonergic signaling in the developing brain has not been systematically investigated.

In this study, we investigated the effects of prolonged MS stress on breast milk composition and examined whether such changes are associated with alterations in offspring GM development, serum metabolic profiles, and serotonergic gene expression in the prefrontal cortex at weaning. Using an integrated multi-omics approach, we aimed to characterize milk-mediated pathways linking maternal stress to offspring neurodevelopment, without presupposing specific microbial taxa or metabolites. We further employed targeted supplementation experiments to explore the functional relevance of candidate milk- and gut-associated metabolites identified through these analyses. To minimize the influence of potential confounders such as litter, sex, circadian rhythm, and diet, these factors were judiciously considered in the study design.

## Results

### MS induces anxiety-related behavior in lactating dams

**Fig. 1:**
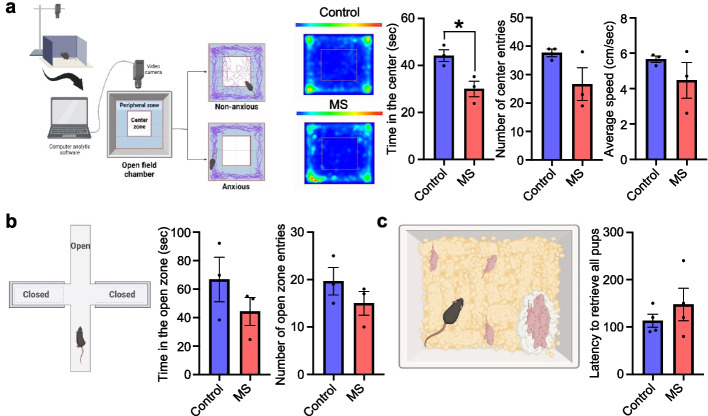
MS induces anxiety-related behavior in lactating dams. Open field test (OFT) results (*n* = 3 per group) showing representative trajectory heat maps and quantification of time spent in the center zone, number of center entries, and average locomotor speed (**a**). Elevated plus maze test (EPMT) results (*n* = 3 per group) showing time spent in the open zone and number of open zone entries (**b**). Pup retrieval test (PRT) results (*n* = 4 per group) showing latency to retrieve all pups (**c**). Data are presented as mean ± SEM. Statistical analyses were performed using Student’s *t*-test (*p < 0.05). Schematic illustrations of the open field test and elevated plus maze test were created with BioRender.com (Agreement no. NM29OGI92A)

An overview of the experimental design and timeline is provided in Fig. S1. To confirm that the MS paradigm induced stress-related behavioral alterations in dams, we assessed anxiety-like and maternal care behaviors using the open field test (OFT), elevated plus maze test (EPMT), and pup retrieval test (PRT). In the OFT, dams subjected to MS spent significantly less time in the center zone compared with control dams (*p* = 0.029), whereas no significant differences were observed in the number of center entries or average locomotor speed (Fig. 1a). In the EPMT, neither the number of entries into the open arms nor the time spent in the open arms differed significantly between MS and control dams (Fig. 1b). In the PRT, the latency to retrieve all pups did not differ between groups (Fig. 1c), indicating that MS induced detectable anxiety-related behavior without gross impairment of pup-directed maternal care.

### MS modestly alters the breast milk metabolome

**Fig. 2:**
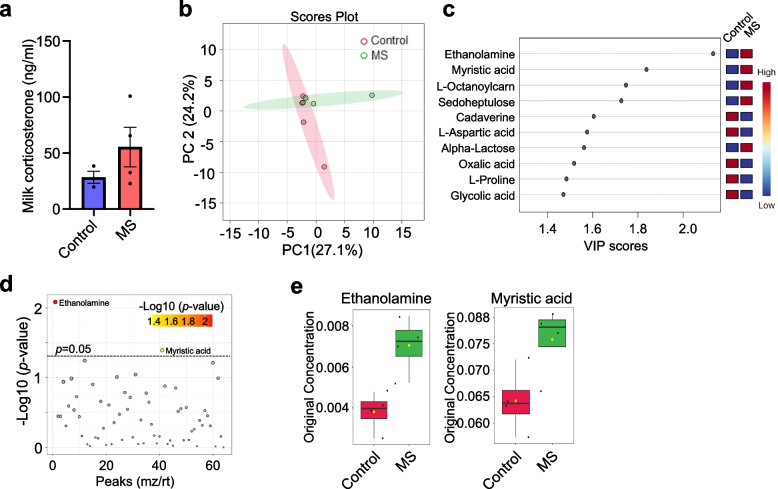
MS modestly alters the breast milk metabolome. Milk corticosterone concentrations in control (*n* = 3) and MS (*n* = 4) dams (**a**). Two-dimensional principal component analysis (PCA) score plot of milk metabolomic profiles (F = 1.27, R^2^ = 0.17, *p* = 0.396) (**b**). Partial least squares discriminant analysis (PLS-DA) of milk metabolomes (*n* = 4 per group) (**c**). Univariate *t*-test identifying significantly altered milk metabolites between groups (**d**). Relative abundance of ethanolamine and myristic acid in milk samples (*n* = 4 per group; ethanolamine *p* = 0.0082; myristic acid *p* = 0.041) (**e**). Data are presented as mean ± SEM. Statistical comparisons were performed without FDR correction as part of an exploratory analysis

To explore whether MS stress was associated with changes in biological signals transmitted via breast milk, we first measured corticosterone concentrations in milk collected from lactating dams. Milk corticosterone levels did not differ significantly between control and MS dams (Fig. 2a). We next performed untargeted gas chromatography-mass spectrometry (GC–MS) based metabolomic profiling of dam milk. A total of 70 metabolites were detected. Principal component analysis (PCA) revealed substantial overlap between control and MS groups, indicating no marked global alteration of the milk metabolome (Fig. 2b). Partial least squares discriminant analysis (PLS-DA) identified several metabolites contributing to group separation based on variable importance in projection (VIP) scores (Fig. 2c). Subsequent univariate analysis revealed that ethanolamine and myristic acid levels were significantly higher in milk from MS dams compared with controls (*p* = 0.008 and *p* = 0.040, respectively) (Fig. 2d, e).

### MS is associated with altered gut microbiota composition in offspring

**Fig. 3:**
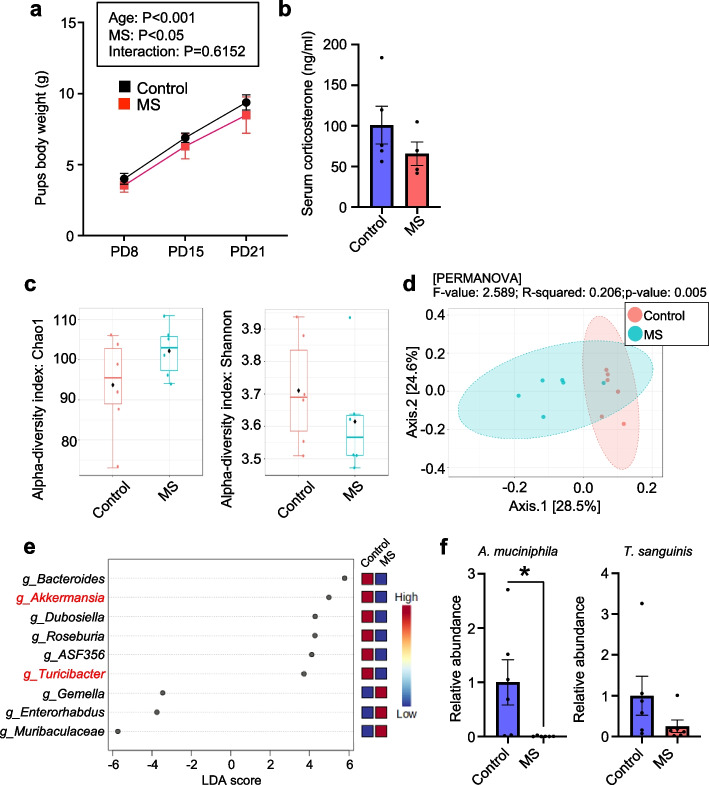
MS is associated with altered gut microbiota composition in offspring. Body weight trajectories of pups on PD8, PD15, and PD21 (two-way repeated-measures ANOVA, *n* = 6 per group) (**a**). Serum corticosterone concentrations in pups after weaning (PD22) (Student’s *t*-test; control *n* = 5, MS *n* = 4) (**b**). Gut microbiota α-diversity indices (Chao1 richness and Shannon diversity) after weaning (PD22) (Welch’s *t*-test, *n* = 6 per group) (**c**). Principal coordinate analysis (PCoA) of gut microbiota β-diversity based on unweighted UniFrac distances (PERMANOVA; F = 2.59, R.^2^ = 0.21, *p* = 0.005; *n* = 6 per group) (**d**). Linear discriminant analysis effect size (LEfSe) identifying differentially abundant genera in pup cecal microbiota (*n* = 6 per group; LDA score > 2.0; *p* < 0.05) (**e**). Quantitative PCR validation of *Akkermansia muciniphila* and *Turicibacter sanguinis* abundance in cecal contents (Student’s *t*-test, *n* = 6 per group) (f). Data are presented as mean ± SEM. *p < 0.05

Given that alterations in systemic growth or stress hormone levels could confound the interpretation of GM analyses, we first examined body weight trajectories and circulating corticosterone levels in offspring exposed to MS. Two-way repeated-measures ANOVA revealed no significant interaction between age and MS, while both age and MS were independently associated with body weight (Fig. 3a). In addition, serum corticosterone levels did not differ significantly between MS and control pups at weaning (Fig. 3b).

Given the role of early milk exposure in shaping microbial colonization, we next examined the GM composition of offspring at weaning. Cecal contents were analyzed using 16S rRNA gene sequencing. α-diversity indices, including Chao1 richness and Shannon diversity, did not differ significantly between MS and control pups (*p* = 0.1695 and *p* = 0.3269, respectively) (Fig. 3c). In contrast, β-diversity analysis based on unweighted UniFrac distances after TSS normalization revealed a significant separation between groups (Permutational multivariate analysis of variance, F-value: 2.589; R^2^: 0.206; *p* = 0.005), indicating differences in overall microbial community composition associated with MS exposure (Fig. 3d). Read count values and Rarefaction curves of different gut microbiota samples are presented in Fig. S2. Linear discriminant analysis effect size (LEfSe) identified several bacterial genera whose relative abundance differed between groups. The genera *Akkermansia*, *Bacteroides*, *Roseburia*, and *Turicibacter* were reduced in MS pups, whereas *Gemella* and *Enterorhabdus* were increased compared with controls (*p* < 0.05) (Fig. 3e, Fig. S3).

To validate microbiota alterations suggested by sequencing analyses, we quantified selected bacterial taxa using quantitative PCR. The abundance of *Akkermansia muciniphila* was significantly lower in the cecal contents of MS pups compared with control pups (*p* = 0.038) (Fig. 3f). Melting curve analysis and gel electrophoresis of PCR amplicons after amplification of *Akkermansia muciniphila* gene-specific DNA fragments are presented in Fig. S4. In contrast, the abundance of *T. sanguinis* did not differ significantly between groups (*p* = 0.1168) (Fig. 3f).

### MS is associated with altered serum and cecal metabolite profiles in offspring

**Fig. 4:**
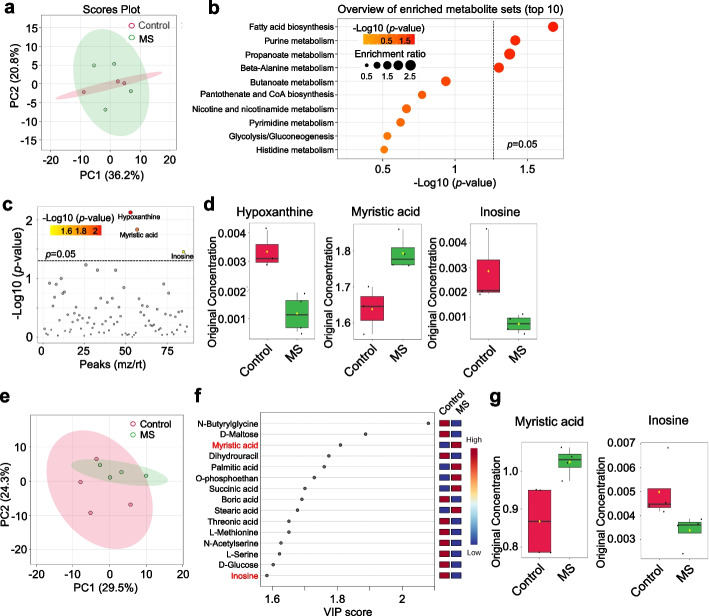
MS is associated with altered serum and cecal metabolite profiles in offspring. Principal component analysis (PCA) score plot of serum metabolomes (F = 0.039, R^2^ = 0.007, *p* = 0.968) (**a**). Kyoto Encyclopedia of Genes and Genomes (KEGG) pathway enrichment analysis showing the top enriched metabolic pathways between control and MS pups (**b**). Univariate *t*-test identifying significantly altered serum metabolites (**c**). Relative concentrations of hypoxanthine, myristic acid, and inosine in serum (control *n* = 3, MS *n* = 4; hypoxanthine *p* = 0.007; myristic acid *p* = 0.014; inosine *p* = 0.034) (**d**). PCA score plot of cecal content metabolomes (F = 1.85, R^2^ = 0.23, *p* = 0.246) (**e**). PLS-DA of cecal metabolomes (*n* = 4 per group) (**f**). Relative abundance of myristic acid and inosine in cecal contents (*n* = 4 per group; myristic acid *p* = 0.022; inosine *p* = 0.061) (**g**). Data are presented as mean ± SEM. Statistical comparisons were performed without FDR correction as part of an exploratory analysis

To assess whether gut-associated alterations were accompanied by serum metabolic changes, we performed untargeted serum metabolomic profiling of pups at weaning. A total of 94 metabolites were detected. PCA revealed substantial overlap between control and MS groups, suggesting no major global shift in serum metabolomic profiles (Fig. 4a). Kyoto Encyclopedia of Genes and Genomes (KEGG) pathway enrichment analysis identified several pathways potentially associated with MS exposure, including fatty acid biosynthesis, purine metabolism, propanoate metabolism, and β-alanine metabolism (Fig. 4b). Univariate analysis revealed that hypoxanthine and inosine levels were significantly reduced in MS pups compared with controls (*p* = 0.007 and *p* = 0.034, respectively), whereas myristic acid levels were significantly elevated in MS pups (*p* = 0.014) (Fig. 4c, d).

Untargeted metabolomic analysis of cecal contents identified 130 metabolites. PCA showed overlapping profiles between groups, indicating no marked global difference (Fig. 4e). PLS-DA highlighted several metabolites contributing to group separation (Fig. 4f). Consistent with serum findings, myristic acid levels were higher in MS pups, whereas inosine levels were lower compared with control pups (Fig. 4g).

### MS is associated with altered serotonergic gene expression in the prefrontal cortex

**Fig. 5:**
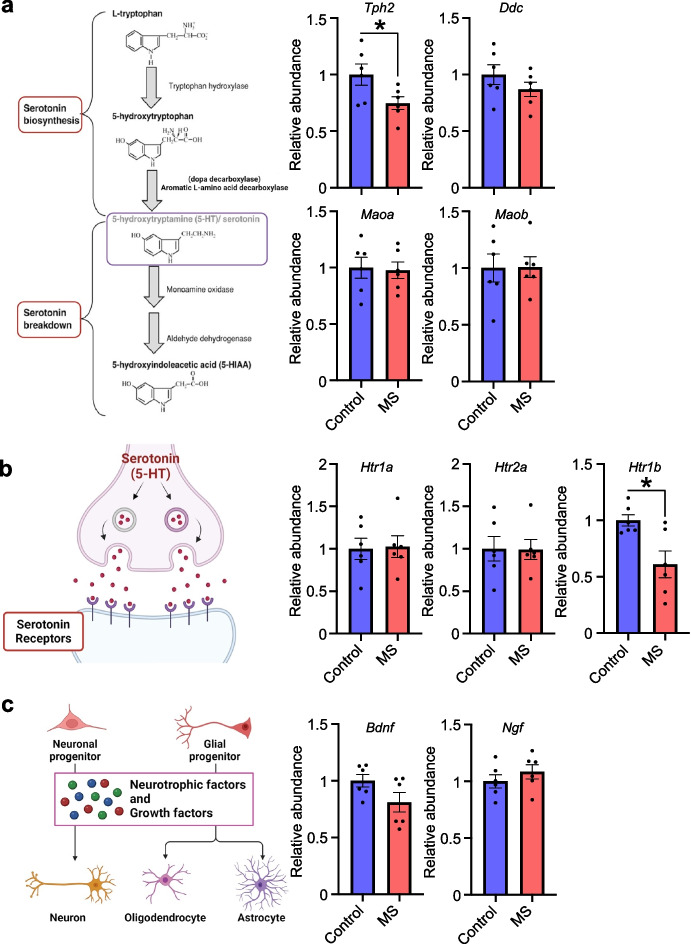
MS is associated with altered serotonergic gene expression in the prefrontal cortex. Reverse transcription–quantitative PCR (RT-qPCR) analysis of genes related to serotonin synthesis and degradation (**a**), serotonin receptor subtypes (**b**), and neurodevelopment-related factors (**c**) in the prefrontal cortex of pups at weaning. Data are presented as mean ± SEM (Student’s *t*-test, *n* = 6 per group). *p < 0.05. Schematic illustrations of serotonin biosynthesis, serotonin receptors, and neurotrophic factors were created with BioRender.com (Agreement no. PQ29OGILPG)

To determine whether MS-associated alterations in GM and serum metabolite profiles were accompanied by changes in brain signaling pathways, we examined the expression of serotonin-related genes in the prefrontal cortex of offspring at weaning. MS was associated with altered expression of metabolic enzyme genes related to serotonin synthesis in the prefrontal cortex. The MS pups exhibited significantly (*p* = 0.043) lower tryptophan hydroxylase 2 (*Tph2*) expression in the prefrontal cortex than control pups (Fig. 5a). Conversely, there was no significant difference in DOPA decarboxylase (*Ddc*) expression between the control and MS groups (Fig. 5a). Likewise, the expression of serotonin breakdown-related gene monoamine oxidase (*Maoa* and *Maob*) did not differ significantly between the two groups (Fig. 5a). Evaluation of the effect of MS on the expression level of serotonin receptor-related genes in the prefrontal cortex revealed that expression of 5-hydroxytryptamine receptor 1B (*Htr1b*) was significantly (*p* = 0.013) lower in the MS group than in the control group. Meanwhile, the expression of other 5-HT receptor subtypes, 1 A (*Htr1a*) and 2 A (*Htr2a*), in the prefrontal cortex did not differ significantly between the MS and control pups (Fig. 5b). Regarding neurodevelopment-related gene expression in the prefrontal cortex, no marked differences were detected in the expression of brain-derived neurotrophic factor and nerve growth factor between the experimental groups (Fig. 5c).

### Myristic acid and inosine supplementation provide partial support for the proposed associations

**Fig. 6:**
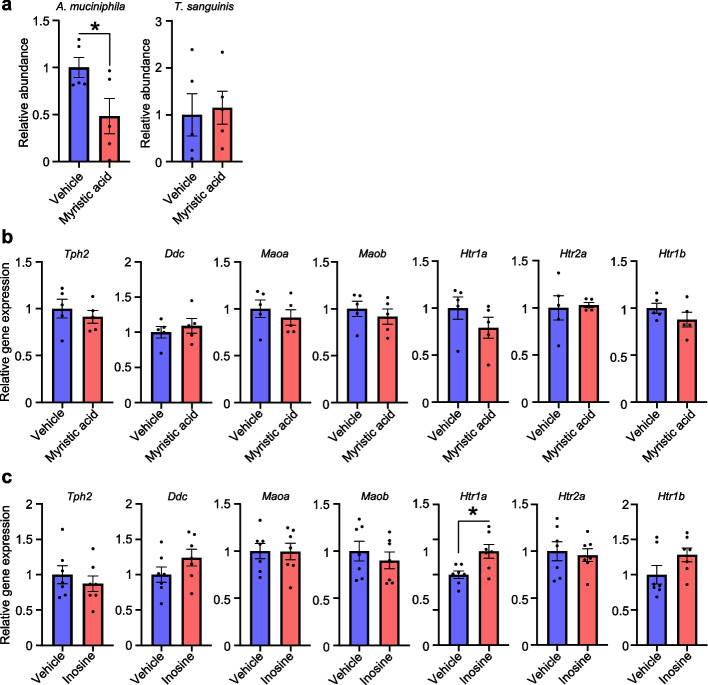
Myristic acid and inosine supplementation provide partial support for the proposed associations. Quantitative PCR analysis of *Akkermansia muciniphila* and *Turicibacter sanguinis* abundance in cecal contents of 2-week-old pups following oral myristic acid administration (**a**). RT-qPCR analysis of serotonin-related gene expression in the prefrontal cortex after myristic acid treatment (**b**). RT-qPCR analysis of serotonin-related gene expression in the prefrontal cortex following inosine administration (**c**). Data are presented as mean ± SEM (Student’s *t*-test, *n* = 5 per group). *p < 0.05

Based on the identification of myristic acid as a milk- and gut-associated metabolite altered by MS, we next examined the effects of oral myristic acid administration in neonatal pups. Myristic acid supplementation was accompanied with a significant reduction in *A. muciniphila* abundance compared with vehicle-treated controls (*p* = 0.044) (Fig. 6a). In contrast, *T. sanguinis* abundance did not differ between groups (Fig. 6a). Myristic acid administration did not significantly alter the expression of serotonin-related genes in the prefrontal cortex (Fig. 6b).

To assess the potential functional relevance of inosine, which was reduced in MS pups, we examined the effects of inosine supplementation on brain gene expression. Inosine-treated pups exhibited a significant increase in *Htr1a* expression in the prefrontal cortex compared with vehicle-treated controls (*p* = 0.0442) (Fig. 6c). No significant changes were observed in the expression of other serotonin-related genes, including *Tph2*, *Ddc*, *Maoa*, *Maob*, *Htr2a*, or *Htr1b*.

### Correlation analyses reveal quantitative associations across multi-omics datasets

**Fig. 7:**
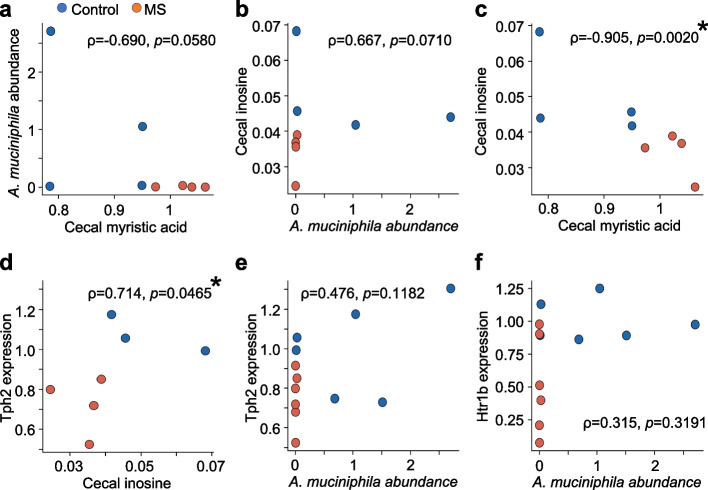
Correlation analyses reveal quantitative associations across multi-omics datasets. Correlation analyses were performed using paired data from the same individual pups to explore quantitative associations among cecal metabolites, gut microbiota, and brain gene expression. **a** Cecal myristic acid levels versus *Akkermansia muciniphila* relative abundance (*n* = 8). **b** *A. muciniphila* relative abundance versus cecal inosine levels (*n* = 8). **c** Cecal myristic acid levels versus cecal inosine levels (*n* = 8). **d** Cecal inosine levels versus *Tph2* relative expression in the prefrontal cortex (*n* = 8). **e** *A. muciniphila* relative abundance versus *Tph2* relative expression in the prefrontal cortex (*n* = 12). **f** *A. muciniphila* relative abundance versus *Htr1b* relative expression in the prefrontal cortex (*n* = 12). Panels a–d represent the subset of animals for which both cecal metabolomic and qPCR data were available (*n* = 4 per group). Panels e and f include all animals with paired microbiota and brain gene expression data (*n* = 6 per group). Blue circles, Control; red circles, MS. Dashed lines indicate linear trend lines. Spearman correlation coefficients (ρ) and p values are shown above each panel. *p < 0.05

To explore quantitative relationships among the datasets, Spearman rank correlation analyses were performed using paired data from the same individual pups. Among pups with both cecal metabolomic and microbiota data (*n* = 8), cecal myristic acid levels were negatively correlated with *A. muciniphila* abundance (ρ =  − 0.690, *p* = 0.058) and with cecal inosine levels (ρ =  − 0.905, *p* = 0.002) (Fig. 7a, c). *A. muciniphila* abundance showed a positive trend with cecal inosine levels (ρ = 0.667, *p* = 0.071) (Fig. 7b). Cecal inosine levels were positively correlated with *Tph2* expression in the prefrontal cortex (ρ = 0.714, *p* = 0.047) (Fig. 7d). When all pups with paired microbiota and brain gene expression data were included (*n* = 12), *A. muciniphila* abundance showed a positive trend with *Tph2* expression (ρ = 0.476, *p* = 0.118) and a non-significant positive trend with *Htr1b* expression (ρ = 0.315, *p* = 0.319) (Fig. 7e, f). These exploratory correlation analyses should be interpreted with caution given the small sample sizes.

## Discussion

Early postnatal life is a critical period for brain development, during which external stressors can shape neurodevelopmental trajectories [3]. MS has been widely employed as a model of early-life stress and is known to influence the gut–brain axis. While maternal care is recognized as a key determinant of offspring neurodevelopment, the contribution of breast milk as a biological conduit for stress-related signals remains insufficiently characterized. In the present study, we employed an integrated multi-omics approach to investigate whether MS-induced changes in breast milk composition are associated with alterations in offspring gut microbiota, metabolite profiles, and serotonin-related gene expression. Importantly, this study was designed as an exploratory, hypothesis-generating analysis, and all findings should be interpreted as associations rather than established causal relationships. Our analyses revealed that MS dams exhibited elevated myristic acid in breast milk, and their offspring showed reduced *A. muciniphila* abundance, decreased inosine levels, and reduced *Tph2* and *Htr1b* expression. Correlation analyses using paired individual data further supported these associations, revealing that cecal myristic acid was negatively correlated with both *A. muciniphila* abundance and cecal inosine, and that cecal inosine was positively correlated with prefrontal *Tph2* expression. These findings provide the first integrative evidence linking maternal stress-induced milk fatty acid changes to offspring gut microbiota, microbial metabolites, and serotonin-related gene expression.

In this study, MS had a limited impact on anxiety-like behavior in dams, as reflected by a reduction in time spent in the center zone of the open field test, while other behavioral indices remained unchanged. These findings are consistent with previous reports describing modest or variable effects of MS on maternal anxiety-like behavior [5, 22]. Pup retrieval test results further indicated that MS did not substantially impair overt maternal care, in agreement with earlier studies reporting unchanged pup retrieval behavior following MS exposure [23]. Given the heterogeneity of reported maternal behavioral outcomes in the MS model [5, 24], these results suggest that maternal-derived factors other than gross behavioral alterations may contribute to offspring phenotypes.

Our metabolomic analysis revealed that myristic acid and ethanolamine were elevated in the breast milk of MS dams. Fatty acids are a major source of energy and bioactive molecules for developing infants, and early-life fatty acid composition has been implicated in long-term health outcomes [25–27]. Previous studies have reported associations between specific fatty acids in human breast milk and maternal cortisol levels, with myristic acid showing a strong positive correlation with cortisol [17]. Although milk corticosterone levels were not significantly altered in the present study, the observed increase in myristic acid suggests that maternal stress may subtly influence milk fatty acid composition, consistent with the notion that stress-related maternal physiology can modify milk-derived metabolic signals even in the absence of overt hormonal changes [18]. Myristic acid was prioritized for functional follow-up because it was consistently elevated across multiple biological compartments (dam milk, pup cecal contents, and pup serum), fatty acid biosynthesis was the top enriched pathway in pup serum KEGG analysis, and saturated fatty acids have well-documented antimicrobial activity. Ethanolamine, although significantly elevated in MS milk, was not detected as differentially abundant in offspring samples; however, its known roles in bacterial signaling and membrane metabolism warrant investigation in future studies.

Microbiota analyses demonstrated no significant differences in α-diversity between MS and control pups, consistent with earlier reports of stable microbial richness following chronic MS exposure [28]. In contrast, β-diversity analyses revealed significant differences in overall microbial community composition, in line with previous observations in MS models involving early-life perturbations [29]. LEfSe analysis identified several genera whose relative abundance differed between groups, including reductions in *Akkermansia*, *Bacteroides*, *Roseburia*, and *Turicibacter* in MS pups. These taxa have been previously associated with intestinal homeostasis and host metabolic health [21]. Targeted quantitative PCR further confirmed a reduction in *Akkermansia muciniphila* abundance in MS pups, whereas *T. sanguinis* was unchanged. Together, these findings indicate that MS is associated with specific alterations in GM composition rather than a global disruption of microbial diversity.

Among serotonergic genes, *Tph2* expression was reduced in the prefrontal cortex of MS pups. As *Tph2* encodes the rate-limiting enzyme for brain serotonin synthesis, its reduced expression may be associated with altered serotonergic regulation during a critical developmental window. Variants in *Tph2* have been linked to mood-related disorders in both humans and animal models [30, 31]. In addition, *Htr1b* expression was reduced in MS pups, consistent with clinical and experimental evidence implicating this receptor in mood regulation and stress-related behaviors [32]. Given that GM and their metabolites are known to influence serotonergic pathways via the gut–brain axis [33], these gene expression changes may represent neurobiological correlates of MS exposure. However, these associations do not establish causal pathways, and the experimental approaches needed to test causality are discussed in the Limitations section.

Serum metabolomic analysis revealed alterations in hypoxanthine and inosine levels in MS pups. Both hypoxanthine and inosine are purine metabolites, and reduced levels of purine pathway metabolites have been reported in depressive states [34]. Inosine is capable of crossing the blood–brain barrier and has been implicated in neural plasticity and neuromodulation [35, 36]. Decreased inosine levels in MS pups may therefore reflect broader metabolic alterations associated with early-life stress [37]. In addition, inosine production has been linked to *A. muciniphila* abundance and gut barrier function [38], suggesting a potential association between microbial shifts and serum metabolite availability. Consistent with this, our correlation analyses revealed that cecal myristic acid levels were negatively correlated with both *A. muciniphila* abundance (ρ =  − 0.690, *p* = 0.058) and cecal inosine levels (ρ =  − 0.905, *p* = 0.002), and *A. muciniphila* abundance showed a positive trend with cecal inosine (ρ = 0.667, *p* = 0.071). These quantitative associations support a coordinated relationship among myristic acid, *A. muciniphila*, and inosine within the gut.

Oral administration of myristic acid was accompanied by a reduction of *A. muciniphila* abundance in neonatal pups, consistent with the known antimicrobial properties of fatty acids [19, 20, 39, 40]. The dose used in this study (1000 mg/kg/day) was selected based on previous reports employing oral fatty acid administration in juvenile or adult mice and was intended as an upper-bound exploratory dose [41, 42]. Although this dose exceeds estimated physiological exposure through breast milk, no adverse effects or overt toxicity were observed. However, the effects on *A. muciniphila* abundance were more modest than those observed in MS pups, and serotonergic gene expression changes were not reproduced. The more pronounced suppression of *A. muciniphila* in MS pups may explain why serotonin-related gene expression changes were observed only in the MS group, although this relationship should be interpreted as associative rather than causative. The limited efficacy of bolus myristic acid administration may reflect differences in bioavailability, as milk-derived fatty acids are incorporated into fat globules and micelles that facilitate efficient intestinal absorption and sustained exposure. Future studies should consider delivering myristic acid within a milk-based matrix or employing dose–response designs to better approximate natural milk-derived exposure conditions.

In contrast, inosine supplementation selectively increased *Htr1a* expression in the prefrontal cortex, while the MS-associated changes in *Tph2* and *Htr1b* were not reproduced. Inosine has been reported to modulate serotonin release and exert antidepressant-like effects in experimental models [34–37, 43]. Although correlation analysis in the main MS experiment revealed a positive association between cecal inosine and *Tph2* expression (ρ = 0.714, *p* = 0.047), the inosine supplementation experiment did not recapitulate this relationship. Inosine was administered subcutaneously based on the observed reduction in serum inosine levels in MS pups. In addition, the use of non-stressed pups further limits direct comparison with the MS model. These findings suggest that while inosine can modulate specific serotonin-related genes, it is insufficient to account for the full spectrum of MS-associated serotonergic changes. Critical next steps would include measuring inosine levels following myristic acid supplementation, and administration of inosine to MS pups to determine whether restoring inosine levels can prevent or reverse MS-associated serotonergic gene expression changes.

Several limitations should be acknowledged. First, regarding study design, only male offspring were analyzed, limiting generalizability given known sex differences in stress responsiveness. Outcomes were assessed only at weaning, and whether these changes persist into adulthood remains to be determined. The current design does not distinguish between direct effects of separation on pups and indirect effects mediated through altered milk composition; cross-fostering experiments would be required to disentangle these pathways. Corticosterone was measured only in dam milk and pup serum under anesthesia, which may not accurately reflect baseline conditions. Second, establishing causal relationships would require germ-free or antibiotic-treated mouse models, fecal microbiota transplantation, *A. muciniphila* supplementation, and in vitro experiments examining the direct effects of myristic acid on *A. muciniphila* growth and metabolite production. Third, regarding analytical scope, we did not profile the breast milk microbiota, and neurobiological assessment was limited to mRNA expression without protein-level validation or direct neurotransmitter quantification. Statistical comparisons were performed without FDR correction, and post hoc power analysis indicated low statistical power (0.149–0.248), well below the conventional threshold of 0.80. Although selecting one randomly chosen male pup per litter avoids pseudo-replication, whether a single pup adequately represents intra-litter variability remains uncertain. These findings should be considered preliminary and require replication in adequately powered cohorts. Finally, direct extrapolation from this rodent model to human contexts must be made with extreme caution, as these findings are preclinical and do not support clinical intervention. The presence of alternative caregivers in human settings may buffer the biological effects of maternal separation, an ecological variable not captured by the current model.

## Conclusions

**Fig. 8:**
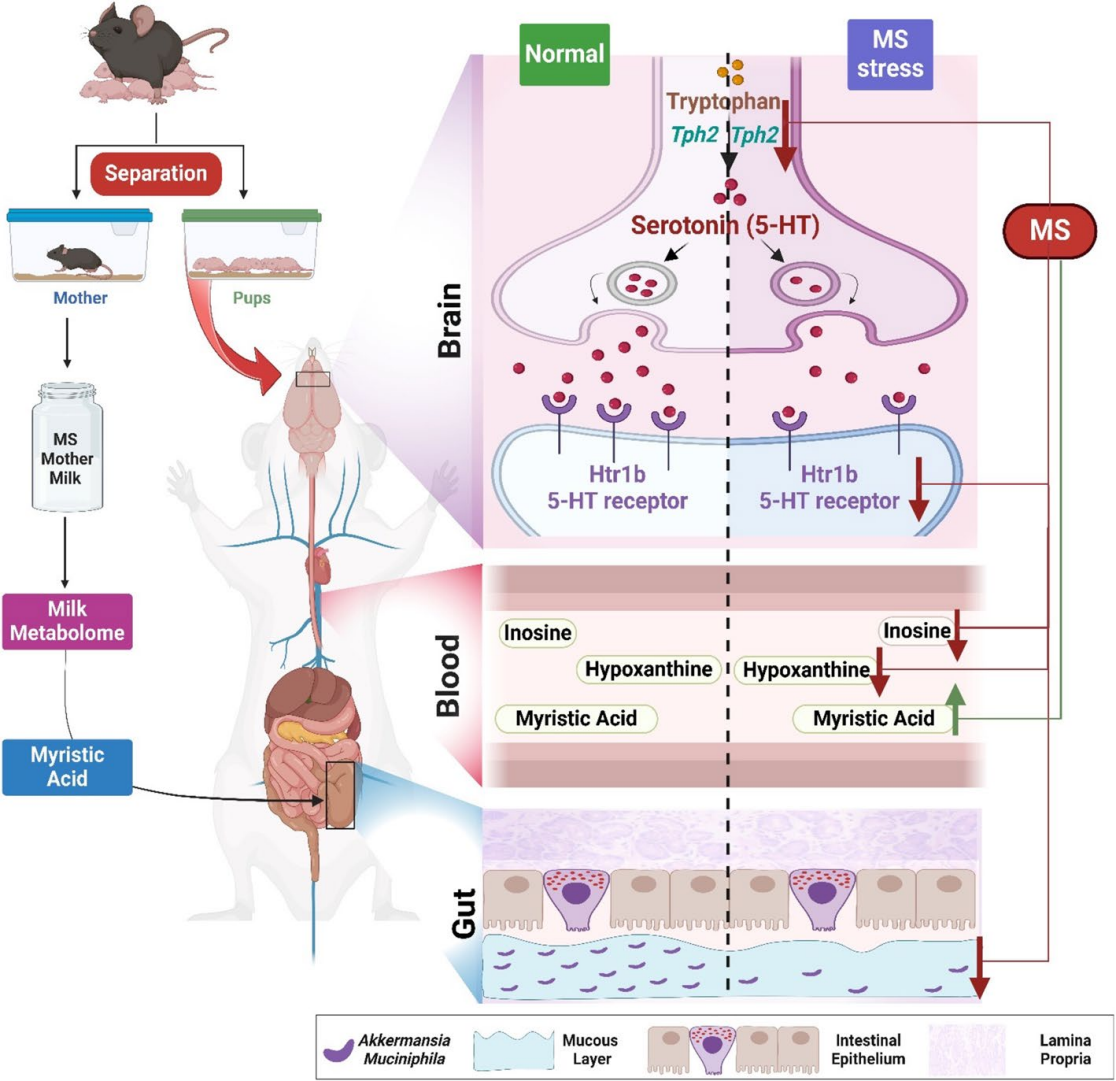
Schematic summary illustrating the coordinated associations identified in this study. Maternal separation is associated with changes in the dam milk metabolome, including increased myristic acid, which coincides with a reduced abundance of *Akkermansia muciniphila* in the offspring gut. These microbial changes are accompanied by alterations in circulating metabolites, such as decreased inosine and hypoxanthine levels, as well as reduced expression of serotonergic genes (e.g., *Tph2* and *Htr1b*) in the prefrontal cortex. The diagram represents an integrative, exploratory framework summarizing observed associations rather than definitive causal pathways. Created with BioRender.com (Agreement no. OY29OGIQK0)

In conclusion, this study identifies coordinated associations among MS stress, breast milk fatty acid composition, offspring GM, serum metabolites, and serotonergic gene expression during early postnatal development (Fig. 8). Notably, quantitative correlation analyses demonstrated that these components are not merely co-occurring but statistically interrelated, with cecal myristic acid, *A. muciniphila* abundance, inosine, and prefrontal *Tph2* expression forming a coherent associative network. These findings underscore the importance of maternal-derived factors in shaping early neurobiological environments through the gut–brain axis, and offer a foundation for future mechanistic studies, including germ-free and cross-fostering models, as well as human translational investigations to isolate the biological effects of milk composition from the direct psychological impact of maternal absence.

## Materials and methods

### Experimental animals and housing

This study was conducted using pregnant C57BL/6J mice (14 weeks old) purchased from CLEA Japan (Tokyo, Japan). Animals were maintained under controlled environmental conditions (14:10 h light/dark cycle; lights on at 05:00, off at 19:00; temperature 23°C ± 2 °C) with ad libitum access to standard chow and tap water. All mice were housed individually from late pregnancy onward to allow accurate monitoring of maternal behavior, milk collection, and pup assignment. Parturition day was designated as postnatal day (PD) 0.

All experimental procedures were approved by the Institutional Animal Care and Use Committee of Tokyo University of Agriculture and Technology (approval no. R04-105) and were performed in accordance with institutional and national guidelines.

### Study design and experimental groups

A total of 20 pregnant dams were used. Twelve dams were assigned to the pup-focused experiments, and eight dams were used exclusively for maternal behavioral and milk analyses to avoid potential confounding effects of repeated handling during pup experiments. Within each set, dams were randomly assigned to either the control group or the MS group. For pup-based analyses (GM, serum metabolome, and brain gene expression), only one male pup was collected from each litter, such that each data point represented an independent litter. This design avoided litter-level pseudo-replication, and therefore no additional statistical correction for litter effects was required. Pups were weaned on PD21. Selected male pups were euthanized on PD22 for sample collection (cecal contents, serum, and brain tissue). Due to the limited sample size, post hoc power analysis using G*Power software (v3.1.9.7) indicated statistical power values of 0.149 and 0.248 to detect effect sizes (Cohen’s d) of 0.761 and 0.813 in dam- and pup-based experiments, respectively, at an α level of 0.05.

### Maternal separation (MS) procedure

The MS model was generated with minor modifications from established protocols [2]. From PD3 to PD20, dams in the MS group were separated from their pups daily for 3 h (11:00–14:00) during the light phase. During separation, pups were placed individually in temperature-controlled containers (32 °C) to prevent hypothermia [12, 44, 45]. From PD15 onward, when pups became more mobile, standard cages were used instead of containers. After each separation period, pups were returned to their home cages and reunited with their dams. Control litters remained undisturbed except for routine body weight measurements.

### Assessment of maternal behavior

Maternal behavior was evaluated to assess the impact of MS stress on dams. All behavioral tests were conducted during the light phase, and experimenters were blinded to group assignment during data acquisition and analysis [46, 47]. Testing equipment was cleaned with 70% ethanol between animals.

The pup retrieval test (PRT) was performed on PD3. Pups were temporarily removed from the home cage and placed at equidistant locations. Dams were then reintroduced, and the latency required to retrieve all pups to the nest was recorded within a 10 min observation period.

The open field test (OFT) was conducted on PD16 using a square arena (40 × 40 × 30 cm). The center zone (20 × 20 cm) was illuminated at 100 lx. Each dam was allowed to explore the arena for 10 min. Time spent in the center zone, number of center entries, and average movement speed were recorded using ANY-maze software (Muromachi Kikai, Tokyo, Japan).

Anxiety-like behavior was further evaluated using the elevated plus maze test (EPMT) on PD18. The maze consisted of two open arms and two closed arms (5 × 25 cm). Each mouse was placed in the central platform facing a closed arm and observed for 10 min. The number of entries and time spent in open and closed arms were quantified using ANY-maze software.

### Milk collection and corticosterone assay

On PD12, milk samples were collected from lactating dams. Mothers were separated from their pups 4 h before the start of milk collection to allow milk to accumulate in the mammary glands. Mice were anesthetized with an anesthetic mixture: 0.3 mg/kg medetomidine hydrochloride (Domitor®; Nippon Zenyaku Kogyo Co., Ltd., Fukushima, Japan), 4 mg/kg midazolam hydrochloride (Dormicum®; Astellas Pharma Inc., Tokyo, Japan), and 5 mg/kg butorphanol tartrate (Vetorphale®; Meiji Seika Pharma Co., Ltd., Tokyo, Japan) via intraperitoneal injection. Oxytocin 10% (O3251-1000IU, Batch No.: SLBM4784V, 567 IU/mg, Sigma Aldrich, St. Louis, MO 63103, USA) solution was prepared by dissolving of oxytocin in normal saline solution (100 µg oxytocin: 900 µL normal saline). Then, each mother was administered 100 µL oxytocin 10% intraperitoneally to induce milk let-down, followed by gentle manual suction using a Dry Aspirator (DAS-01, AS ONE Corporation, Japan). Following the collection of milk samples, 100 μl milk obtained from each lactating mother was two-fold diluted with 100 μl ultra-pure water and then was centrifuged (21,500 × g, 60 min, 4 °C). The middle layer (skim milk) was aspirated and immediately frozen at −80 °C until analysis.

Corticosterone concentrations in dam milk and pup serum were measured using a commercial ELISA kit (Enzo Life Sciences, Japan) according to the manufacturer’s instructions. Milk and serum samples were diluted 1:40 and 1:400, respectively.

### Sample collection from pups

On PD22, selected male pups were anesthetized with the same anesthetic mixture described above. Blood was collected, after which animals were euthanized by decapitation. Serum was separated by centrifugation, pretreated by diluting fivefold using ultrapure water, and stored at −80 °C. Cecal contents were collected under sterile conditions and immediately frozen. For metabolomic analysis, 30 mg of cecal contents were suspended using 120 μl ultrapure water (Thermo Fisher Scientific). After centrifugation (20,000 × g, 4 °C, 5 min), the supernatant was collected and stored at −80 °C. Brains were rapidly removed, and the prefrontal cortex was dissected on ice, snap-frozen, and stored at −80 °C.

### DNA extraction and 16S rRNA gene sequencing

Genomic DNA was extracted from cecal contents using the QuickGene DNA Tissue Kit (Kurabo Industries, Osaka, Japan) [48]. Approximately 25 mg of cecal content was homogenized with glass beads, followed by enzymatic digestion and purification according to the manufacturer’s protocol.

The V3–V4 regions of the bacterial 16S rRNA gene were amplified using primers 341 F and 805R [12, 47]. Amplicon libraries were prepared with dual indices and sequenced using the Illumina MiSeq platform with the MiSeq v3 reagent kit. Negative extraction controls were included. Sequence data were processed using DADA2 (version 1.14) within QIIME2 (version 2021.2), including quality filtering, chimera removal, and amplicon sequence variant (ASV) inference [49]. Taxonomic assignment was performed using the SILVA reference database (version 138).

### Quantitative PCR for target bacteria

Quantitative PCR (qPCR) was used to quantify the abundance of *Akkermansia muciniphila* and *Turicibacter sanguinis* in cecal DNA samples. Primer sequences are provided in Table S1. Amplification specificity was confirmed by melt-curve analysis.

### RNA extraction and RT-qPCR of brain tissue

Total RNA was extracted from prefrontal cortex samples using ISOGEN II reagent (Nippon Gene, Tokyo, Japan). Complementary DNA was synthesized using PrimeScript™ II 1 st Strand cDNA Synthesis Kit (Takara Bio, Japan). Quantitative PCR was performed using PowerUp™ SYBR™ Green Master Mix (Applied Biosystems). Relative gene expression was calculated using the 2 − ΔΔCt method, with β-tubulin as the reference gene [12]. Primer sequences are listed in Table S2.

### Metabolomic analysis

Untargeted metabolomic analysis of milk, serum, and cecal content samples was performed using gas chromatography-mass spectrometry (GC–MS). This platform primarily detects low-molecular-weight, derivatizable metabolites, including organic acids, amino acids, sugars, and selected fatty acids; large macromolecules such as proteins and complex oligosaccharides are not comprehensively captured.

Sample preparation, derivatization, and GC–MS acquisition were conducted as described previously [48]. 2-Isopropylmalic acid was used as an internal standard. Metabolite identification was based on retention time and mass spectral matching against the Shimadzu Smart Metabolite Database. Data were processed and analyzed using MetaboAnalyst 6.0.

### Myristic acid administration to pups

In a separate experiment, neonatal pups were orally administered myristic acid (˃99% purity; Sigma Aldrich, St. Louis, MI, USA, code no.: 70079-5G) at a dose of 1000 mg/kg body weight once daily from PD3 to PD14. Myristic acid was dissolved in 100 μl of alcohol (Ethanol 99,5% purity; Sigma Aldrich, St. Louis, MI, USA, code no.: 09–0770-4), then dispersed in 10% (wt./vol.) bovine serum albumin (BSA, fatty acid-free; Sigma Aldrich, St. Louis, MI, USA) (100 mg myristic acid dissolved in 100 µL ethanol then dispersed in 900 µL BSA). The pH of the prepared myristic acid solution was estimated using pH testing paper and it was approximately 5. Vehicle-treated control pups received the same ethanol/BSA solution without myristic acid. All animals used for sample collection tolerated the technique and treatment without obvious adverse effects. Pups that did not survive the procedure were excluded from the analysis. On PD14, pups were euthanized, and cecal contents and prefrontal cortex samples were collected. The abundance of *A. muciniphila* and *T. sanguinis* in cecal contents was quantified by qPCR, and serotonin-related gene expression in the prefrontal cortex was measured by RT-qPCR.

### Inosine administration to pups

In another cohort, 14 mice pups were randomly assigned to one of two groups (*n* = 7/group). Inosine was dissolved in normal saline (20 mg/mL). Pups received daily subcutaneous (in the dorsal region of the neck) injections of 10 mL/Kg equivalent to 200 mg/kg inosine (Wako Pure Chemical Industries Osaka, Japan, Cat. no. 097–00232) from PD11 to PD26. PD11 was selected as the starting point for subcutaneous administration because neonatal skin thickness at this stage reduces the risk of retrograde leakage. Treatment continued until PD26 to assess the cumulative effects of inosine during a period of active neurodevelopment [50, 51]. Control animals received equivalent volumes of saline. Pups were euthanized on PD27, and prefrontal cortex samples were collected for gene expression analysis.

### Statistical analysis

All statistical analyses were performed using GraphPad Prism version 9 (GraphPad Software, La Jolla, CA, USA) unless otherwise stated. Data are presented as mean ± standard error of the mean (SEM). The level of statistical significance was set at *p* < 0.05. Because only one male pup was selected from each litter for pup-based experiments, each data point represented an independent biological replicate (litter-level replication), and no correction for litter effects or mixed-effects modeling was required.

Body weight data of pups were analyzed using two-way repeated-measures analysis of variance (ANOVA) with factors of group (Control vs MS) and age (PD), followed by the Benjamini–Hochberg false discovery rate (FDR) correction; where Age, *p* < 0.0001, F (1.223, 12.23) = 192.8, df = 2, MS, *p* = 0.0489, F (1, 10) = 5.024, df = 1, Interaction, *p* = 0.6152, F (1.223, 12.23) = 0.3384, df = 2, 95% CI of difference = 0.003857 to 1.304. Post-hoc details: Control-MS, Day 8, q value = 0.1640, Individual *p* value = 0.0940, Day 15, q value = 0.1640, Individual *p* value = 0.1640, Day 21, q value = 0.1640, Individual *p* value = 0.1627.

For comparisons between two groups, Student’s *t*-test was applied when data met assumptions of normality and homogeneity of variance. When these assumptions were not met, data distribution was inspected visually, and appropriate nonparametric alternatives were considered.

MicrobiomeAnalyst version 2.0 (https://www.microbiomeanalyst.ca/MicrobiomeAnalyst/) was used for statistical analysis with default settings. GM α-diversity indices (Chao1 and Shannon) were compared between groups using Student’s *t*-test. Microbiota β-diversity was assessed by principal coordinate analysis (PCoA) based on unweighted UniFrac distances. Group differences in community composition were tested using permutational multivariate analysis of variance (PERMANOVA) with 999 permutation. Homogeneity of multivariate dispersion was evaluated prior to PERMANOVA to confirm that significant differences were not driven by unequal within-group variance. Differentially abundant taxa were initially explored using linear discriminant analysis effect size (LEfSe) with a logarithmic linear discriminant analysis (LDA) score threshold of 2.0. LEfSe results were considered exploratory and were interpreted in conjunction with relative abundance data and targeted quantitative PCR validation. Given the exploratory nature of the microbiota analyses and the limited sample size, results are reported based on unadjusted *p* values, as commonly applied in hypothesis-generating studies.

For metabolomic analyses, metabolite identification criteria were clarified according to the Metabolomics Standards Initiative (MSI). MSI Level 1 (confirmed identification) was assigned. Hard ionization, positive ion mode, retention index tolerance (RI tolerance = 20), retention time tolerance (RT tolerance = 0.5 min) and MS/MS fragmentation spectra were employed. Data were normalized to the internal standard (2-isopropylmalic acid) and autoscaled prior to statistical testing. Unsupervised principal component analysis (PCA) was used to visualize global metabolic patterns. Supervised partial least squares discriminant analysis (PLS-DA) was employed as a complementary exploratory tool to identify metabolites contributing to group separation. Group differences in individual metabolite levels were evaluated using Student’s *t*-test without false discovery rate (FDR) correction, consistent with the exploratory design of this study. Statistical significance was interpreted cautiously, with emphasis placed on effect directionality, biological plausibility, and consistency across milk, cecal content, and serum datasets. Pathway enrichment analysis was performed using MetaboAnalyst 6.0 based on Kyoto Encyclopedia of Genes and Genomes (KEGG) annotations, using nominal *p* values to identify potentially affected metabolic pathways [52].

Spearman rank correlation analyses were performed to explore associations across multi-omics datasets using paired data from the same individual animals. Spearman rank correlation was selected because the Shapiro–Wilk test indicated that *A. muciniphila* abundance, which is included in all correlation analyses, did not follow a normal distribution (*p* < 0.001). Analyses were conducted using data from pups with both cecal metabolomic and qPCR data (*n* = 8; 4 per group) or all pups with paired microbiota and brain gene expression data (*n* = 12; 6 per group). Correlation coefficients (ρ) and associated p values are reported. Given the small sample sizes, these analyses are considered exploratory.

## Supplementary Information

Supplementary Material 1.

Supplementary Material 2.

## Data Availability

All 16S rRNA sequencing data for gut microbiota were uploaded to NCBI Bioproject and are accessible at PRJNA1148830. All metabolic data were uploaded to MetaboLights and can be located under accession number MTBLS11410. Raw data of microbiome, metabolome and repositories bioprojects are presented at Dataset S1.
