# Supplementary material for: Maternal separation-induced changes in breast milk fatty acid composition are associated with altered gut microbiota and serotonergic gene expression in neonatal mice
Source: Mol Biomed. 2026 Jun 28;7:100. doi: 10.1186/s43556-026-00508-2 (PMC13310859; doi:10.1186/s43556-026-00508-2)
Supplement: Supplementary file 2 — Supplementary Material 2. [file 43556_2026_508_MOESM2_ESM.docx]

**Supplementary Information**

**Maternal separation-induced changes in breast milk fatty acid composition are associated with altered gut microbiota and serotonergic gene expression in neonatal mice.**

Eman A. Mady^a,b^, Hussein M. El-Husseiny^a,c,d^, Jun Kambe^a^, Sora Masukado^a^, Shiho Miyata^a^, Taiki Terajima^a^, Hong Liu^a^, Ryo Inoue^e^, Chunmei Li^f^, Yuki Yamamoto^a^, Kentaro Nagaoka^a^*

^a^ Laboratory of Veterinary Physiology, Cooperative Department of Veterinary Medicine, Tokyo University of Agriculture and Technology, Tokyo 183-8509, Japan.

^b^ Department of Animal Hygiene, Behavior, and Management, Faculty of Veterinary Medicine, Benha University, Moshtohor, Toukh 13736, Elqaliobiya, Egypt.

^c^ Institute of Global Innovation Research, Tokyo University of Agriculture and Technology,

3-8-1 Harumi-cho, Fuchu-shi, Tokyo 183-8538, Japan.

^d^ Department of Surgery, Anesthesiology, and Radiology, Faculty of Veterinary Medicine, Benha University, Moshtohor, Toukh, Elqaliobiya, Egypt

^e^ Laboratory of Animal Science, Department of Applied Biological Science, Setsunan University, Osaka, Japan

^f^ College of Animal Science and Technology, Nanjing Agricultural University, Nanjing, China

*Corresponding author: Kentaro Nagaoka, D.V.M., Ph.D. Laboratory of Veterinary Physiology, Department of Veterinary Medicine, Tokyo University of Agriculture and Technology, Tokyo 183-8509, Japan. Fax: +81 042-367-5767; Tel: +81 042-367-5767; Email: [nagaokak@cc.tuat.ac.jp](mailto:nagaokak@cc.tuat.ac.jp)

Supplementary Figure S1, Eman et al.

**a**

**b**

**b**

**Supplementary Figure S1. Experimental design and study timeline.**

Schematic overview of animal grouping (a) and experimental timeline (b). Maternal separation (MS) was conducted daily from postnatal day (PD) 3 to PD20. Milk samples were collected from dams on PD12. Maternal behavior tests were performed on PD3 (pup retrieval test), PD16 (open field test), and PD18 (elevated plus maze test). Pups were weaned and sexed on PD21 and sacrificed for sample collection on PD22. Created with BioRender.com (Agreement no. AF29OGI44I).

Supplementary Figure S2, Eman et al.

**a**


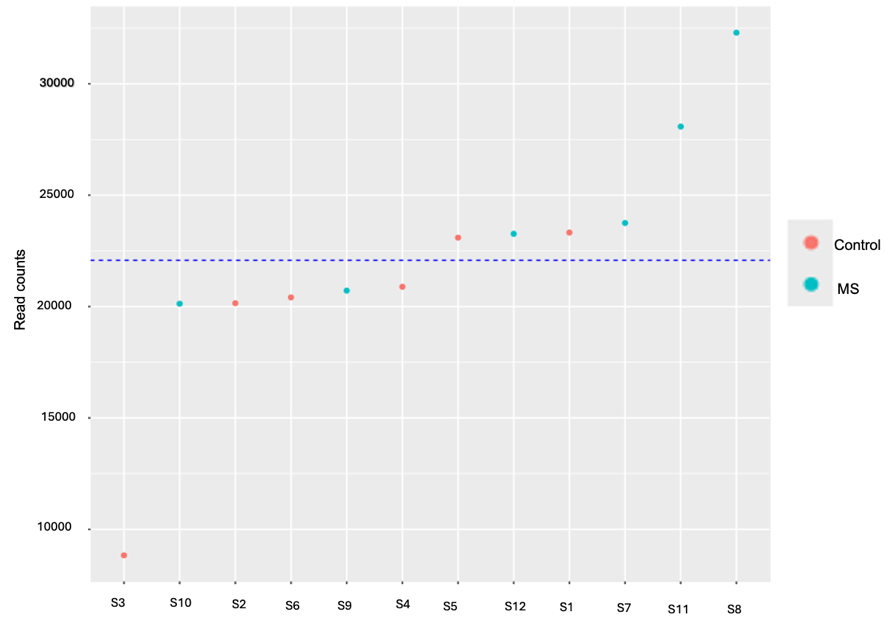


**b**


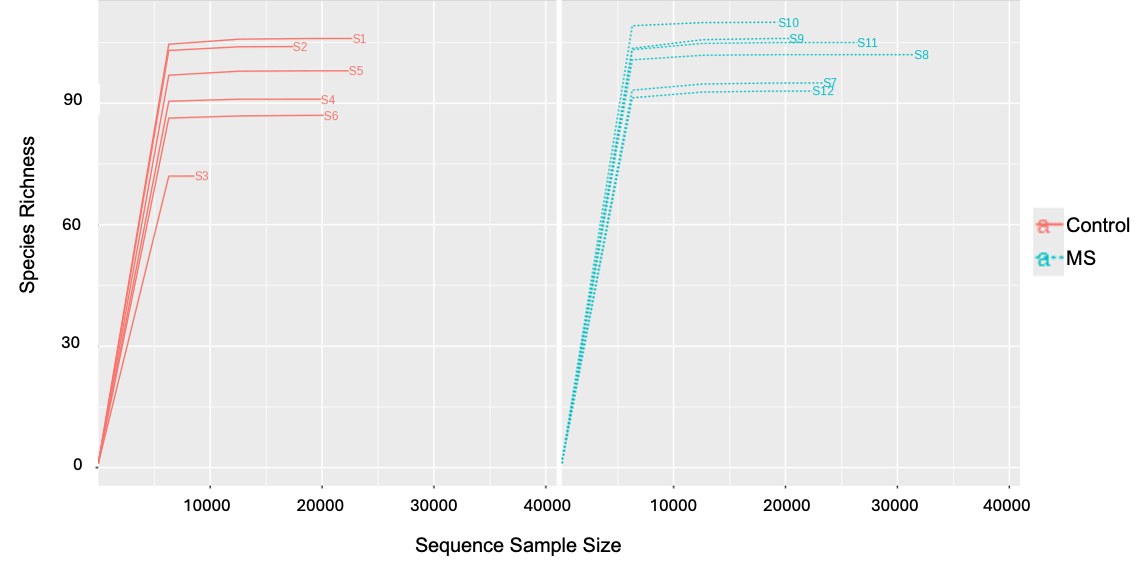


**Supplementary Figure S2.** Read counts values of different gut microbiota samples for different groups in the present study (a). Rarefaction curves to analyze microbiota samples in terms of species richness (b).

Supplementary Figure S3, Eman et al.

#
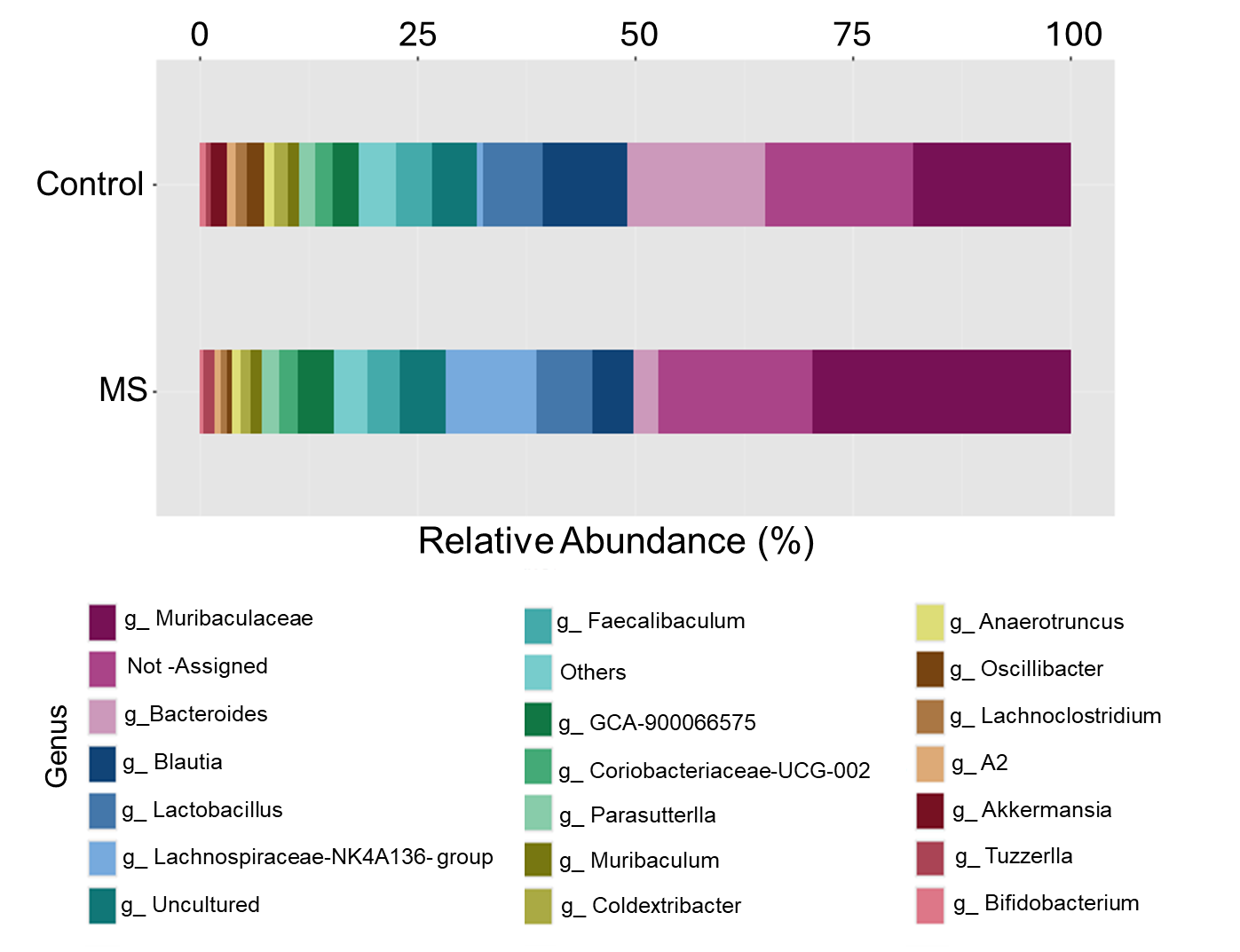


**Supplementary Figure S3.** Relative abundance (%) of bacteria at genus level detected in the gut microbiota of different groups after maternal separation in the present study.

Supplementary Figure S4, Eman et al.


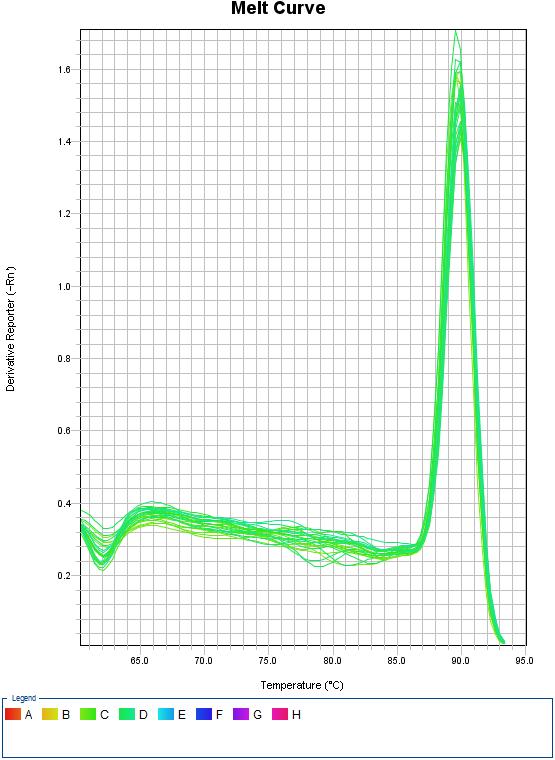


# Supplementary Figure S4. Left panel: Agarose gel electrophoresis of PCR amplicons after amplification of *Akkermansia muciniphila* gene-specific DNA fragments. Right panel: Melting curve analysis from *Akkermansia muciniphila* quantitative PCR assay.

# Supplementary Table S1. Eman et al.

Table S1. Primer sequences for the bacterium used for quantitative polymerase chain reaction **(**qPCR).

| Name | Forward primer sequence (5′ to 3′) | Reverse primer sequence (5′ to 3′) | Size (bp) | Ref. |
| --- | --- | --- | --- | --- |
| *Akkermansia muciniphila.* | CAGCACGTGAAGGTGGGGAC | CCTTGCGGTTGGCTTCAGAT | 327 | [1-4] |
| *Turicibacter sanguinis* | GACGGTACCTTATGAGAAAGC | CACTTCCCTCTTCTGCAC | 193 | This study |
| *Total Bacteria* | ACTCCTACGGGAGGCAGCAGT | GTATTACCGCGGCTGCTGGCAC | 176 | [5-7] |

Supplementary Table S2. Eman et al.

Table S2. Primer sequences for the genes used for quantitative reverse transcriptase polymerase chain reaction **(**qRT-PCR).

| Gene | Forward primer sequence (5′ to 3′) | Reverse primer sequence (5′ to 3′) | Size (bp) | Ref. |
| --- | --- | --- | --- | --- |
| *Tph2* | TTCACAGTGAGACCAGTGGC | CATGGCATGTATCTGGTTCCG | 139 | This Study |
| *Ddc* | CCAGAGTTCACACAAGCTGC | CTACAGAGGAATGCGCCTGA | 73 | This Study |
| *Maoa* | ACCAGAGCTTCCACCTGAGA | TCATGCAGCCACAATAGTCCTT | 122 | This Study |
| *Maob* | CAACCAATGGAGGACAGGAGA | AGCAGGTGGAATGGCACTAAT | 200 | This Study |
| *Htr1a* | GGATGTTTTCCTGTCCTGGT | CACAAGGCCTTTCCAGAACT | 121 | [8] |
| *Htr2a* | AGAACCCCATTCACCATAGC | ATCCTGTAGCCCGAAGACTG | 119 | [8] |
| *Htr1b* | TCACATGGCCATTTTTGACT | CAGTTTGTGGAACGCTTGTT | 112 | [8] |
| *Bdnf* | GCTCAGCAGTCAAGTGCCTTT | CCTTTGGATACCGGGACTTTC | 240 | This Study |
| *Ngf* | CCAAGCTCACCTCAGTGTCTG | TTACGCTATGCACCTCACTGC | 87 | This Study |
| *Tubulin* | GCTGACCAGTGCACGC | AAACCTGGGGGGCTGGGT | 157 | [9] |

**Supplementary Datasets (separate file).**Metabolome and microbiome data analyzed by MetaboAnalyst 6.0 and MicrobiomeAnalyst 2.0 in the present study.

**References:**

[1] M.C. Dao, A. Everard, J. Aron-Wisnewsky, N. Sokolovska, E. Prifti, E.O. Verger, B.D. Kayser, F. Levenez, J. Chilloux, L. Hoyles, M.I.-O. Consortium, M.-E. Dumas, S.W. Rizkalla, J. Doré, P.D. Cani, K. Clément, &lt;em&gt;Akkermansia muciniphila&lt;/em&gt; and improved metabolic health during a dietary intervention in obesity: relationship with gut microbiome richness and ecology, Gut 65(3) (2016) 426. 10.1136/gutjnl-2014-308778

[2] C. Liu, X. Zheng, J. Ji, X. Zhu, X. Liu, H. Liu, L. Guo, K. Ye, S. Zhang, Y.-j. Xu, X. Sun, W. Zhou, H.L.X. Wong, Y. Tian, H. Qian, The carotenoid torularhodin alleviates NAFLD by promoting Akkermanisa muniniphila-mediated adenosylcobalamin metabolism, Nature Communications 16(1) (2025) 3338. 10.1038/s41467-025-58500-3

[3] K. Sonoyama, T. Ogasawara, H. Goto, T. Yoshida, N. Takemura, R. Fujiwara, J. Watanabe, H. Ito, T. Morita, Y. Tokunaga, T. Yanagihara, Comparison of gut microbiota and allergic reactions in BALB/c mice fed different cultivars of rice, British Journal of Nutrition 103(2) (2010) 218-226. 10.1017/S0007114509991589

[4] A. Alam, G. Leoni, M. Quiros, H. Wu, C. Desai, H. Nishio, R.M. Jones, A. Nusrat, A.S. Neish, The microenvironment of injured murine gut elicits a local pro-restitutive microbiota, Nature Microbiology 1(2) (2016) 15021. 10.1038/nmicrobiol.2015.21

[5] A.T. Reese, F.C. Pereira, A. Schintlmeister, D. Berry, M. Wagner, L.P. Hale, A. Wu, S. Jiang, H.K. Durand, X. Zhou, R.T. Premont, A.M. Diehl, T.M. O’Connell, S.C. Alberts, T.R. Kartzinel, R.M. Pringle, R.R. Dunn, J.P. Wright, L.A. David, Microbial nitrogen limitation in the mammalian large intestine, Nature Microbiology 3(12) (2018) 1441-1450. 10.1038/s41564-018-0267-7

[6] A. Fite, S. Macfarlane, E. Furrie, B. Bahrami, H. Cummings John, T. Steinke Douglas, T. Macfarlane George, Longitudinal Analyses of Gut Mucosal Microbiotas in Ulcerative Colitis in Relation to Patient Age and Disease Severity and Duration, Journal of Clinical Microbiology 51(3) (2013) 849-856. 10.1128/jcm.02574-12

[7] A. Bergström, T.R. Licht, A. Wilcks, J.B. Andersen, L.R. Schmidt, H.A. Grønlund, L.K. Vigsnæs, K.F. Michaelsen, M.I. Bahl, Introducing GUt Low-Density Array (GULDA) – a validated approach for qPCR-based intestinal microbial community analysis, FEMS Microbiology Letters 337(1) (2012) 38-47. 10.1111/1574-6968.12004

[8] T. Bibancos, D.L. Jardim, I. Aneas, S. Chiavegatto, Social isolation and expression of serotonergic neurotransmission-related genes in several brain areas of male mice, Genes, Brain and Behavior 6(6) (2007) 529-539. <https://doi.org/10.1111/j.1601-183X.2006.00280.x>

[9] E.A. Mady, H.M. El-Husseiny, Y. Makioka-Itaya, S. Ishikawa, R. Inoue, C. Li, Y. Yamamoto, K. Nagaoka, Therapeutic potential of heat-killed Enterococcus faecalis (EC-12) para-probiotic in reversing maternal separation-triggered social deficit in mice, Biomedicine & Pharmacotherapy 191 (2025) 118513. <https://doi.org/10.1016/j.biopha.2025.118513>
